# Resonance Frequency as an Indicator of the Damage in Carbon Composite Plates: Analysis on Composites Prepared with Conventional and Sustainable Resins Subjected to Impact Tests

**DOI:** 10.3390/polym17020141

**Published:** 2025-01-08

**Authors:** Raffaele Ciardiello, Carlo Boursier Niutta, Andrea Tridello

**Affiliations:** Department of Mechanical and Aerospace Engineering, Politecnico di Torino, 10129 Turin, Italy; carlo.boursier@polito.it

**Keywords:** crash, composite, carbon fibres, sustainable resins, bio resin, damage progression

## Abstract

This paper experimentally investigates the impact response of composite laminates made with conventional and bio-based epoxy resin. Drop tower impact tests were conducted at varying energy levels, including repeated low-energy impacts, to evaluate perforation resistance. The laminates’ residual strength and damage tolerance were assessed using the Damage Index (DI) and by analysing the resonance frequency variations through the Impulse Excitation Technique (IET). The study demonstrates a strong correlation between the DI and the resonance frequencies of the specimens, suggesting that IET can effectively track damage progression in composite laminates. Bio-based resin laminates exhibited higher energy absorption at perforation and lower damage progression during repeated impacts due to the higher ductility of the resin. This method of using resonance frequencies to assess impact damage progression directly in composite laminates throughout the IET technique has not been previously reported in the literature.

## 1. Introduction

Sustainability and circular economy have become essential in many industrial sectors, including automotive and aerospace, driven by stringent regulations targeting reduced CO_2_ emissions [1,2,3,4]. Lightweight design is crucial in achieving these objectives, and composite materials are used due to their high specific strength to reduce the vehicle weight.

### 1.1. Novel Composite Materials with Lower Impact on the Environment

Composite materials, known for their high specific strength, are preferred over metallic materials in lightweight applications [5,6]. Epoxy matrix composites are the most widespread for structural uses but raise sustainability concerns due to their petroleum-based origin, non-recyclability, and significant carbon footprint, contributing over 80% of the total footprint in the automotive sector [7]. To address these issues, there is a shift towards more sustainable composites without compromising structural integrity. While thermoplastic matrices offer recyclability, their lower strength and thermal stability currently limit their use in critical applications [8,9]. Another alternative is to adopt composite materials with high bio-resin content, like [10]. Still, they share the same criticality of a thermoplastic matrix, with lower mechanical properties and lower glass transition temperatures, which are inappropriate for the current in-service applications. However, the research on fully bio composite is active, fuelled by industrial needs and strict regulations imposed on it. An interesting compromise in this transition is represented by an epoxy matrix composite, with the matrix composed of a percentage of petrol-based resin and a percentage of bio-based resin. In this way, the high carbon footprint associated with the epoxy-based matrix can be significantly reduced. However, this mix can affect the structural integrity of the resulting laminates, requiring their mechanical response against typical in-service loads to be carefully verified to ensure their safe use. For example, the percentage of bio-based resin can affect the mechanical response, as shown in [11], with a significant drop of 77% in the elastic modulus and 70% in the tensile strength after increasing the bio content from 27% to 51%. In [12], the quasi-static properties, tensile, shear bending and compression of epoxy-based and bio-based composite materials have been compared with limited differences. Moreover, the research also focuses on the absorption capabilities of composite materials with hybrid fibres since composite materials are largely employed in this field, e.g., crash tubes in the automotive sector [13,14]. For a safe design, the impact properties should be adequately experimentally assessed, focusing on perforating and low-energy repeated impacts, with accumulated damage that may induce final failure. In the latter case, the characterisation of the residual strength of the impacted composite is fundamental to in-service applications and should be experimentally verified. This analysis has proven the potentialities of composite with hybrid matrix, pointing out the need for extensive experimental characterisation to ensure their safe use in structural applications against all loads. Another recent approach to reducing the carbon footprint of composite materials is using vitrimer-based composite materials, which offer enhanced recyclability and repairability. Their dynamic covalent bonds enable easier reprocessing and recycling at the end of their lifecycle, reducing waste and the need for virgin material production and lowering greenhouse gas emissions [15].

### 1.2. Reducing the Carbon Footprint of Composite Materials by Assessing the Residual Elastic Properties for Reusing Components

Reducing the carbon footprint by using raw materials manufactured with more sustainable processes is only one of the strategies that can be adopted to minimise the impact of these materials on the environment. Another strategy is reusing the components at end-of-life or when vehicles are dismantled. However, this strategy requires verifying the components to assess their health state.

In impact testing of composite materials, damage refers to physical and mechanical alterations caused by external forces or energy, which degrade the performance of specimens or components. This damage appears as matrix cracking, delamination, fibre breakage, debonding, surface indentation, and void formation.

Damage assessment in composite materials has been investigated over the last 20 years. As reported by Boursier Niutta et al. [16] in a recent review, different techniques can be used to detect damage assessment in composite laminates. The most used techniques are wave methods (ultrasonic, acousto-ultrasonic, and nonlinear acoustic techniques), vibrational methods, optical methods, thermography, electrical resistivity, and acoustic and computed tomography (CT) methods.

Research indicates that acoustic and CT methodologies are limited to qualitatively assessing damage in composite laminates [17] without offering quantitative indices or parameters related to the elastic properties of the components. In ultrasonics and acousto-ultrasonics [18,19,20], time-of-flight measurements become controversial when there is a significant difference between phase velocity and group velocity, which restricts the application of these techniques to cross-ply laminates. However, using acoustic emission analysis methods, such as evaluating the Stress Wave Factor in the frequency domain, appears highly promising.

On the other hand, global and local vibrational analyses can assess the global or local material properties. The primary limitation of this technique is its applicability only to planar or partially curved surfaces [21,22,23]. However, these vibrational techniques, being a novel method, require further investigation and validation.

Optical methods [24,25], such as Digital Image Correlation and interferometric techniques, can capture local and global deformations. This methodology can depict local displacement gradients, thus allowing for local material characterisation. However, this methodology needs the application of an external load, which might limit its application to real-world components. Further, a speckle pattern may be applied, but this is not possible or can be difficult in components that have to be reused.

Thermography techniques [26,27] can be used to inspect significant components in a relatively short time. However, the non-mechanical nature of these techniques limits their application for quantitative damage assessment. Specifically, they cannot account for the directional dependency of material properties. Additionally, it is essential to note that temperature is not an inherent material property in thermography.

Among these methodologies, novel resistivity techniques, which use conductive fillers or piezoelectric sensors in composites, are also worth mentioning to monitor the electrical resistance of materials related to damage progression [28,29]. Although this technique looks promising, a preliminary study of its reliable applicability is needed.

The above analyses have proven the importance of reliably assessing the residual properties of composite materials subjected to random loads. It has highlighted the strengths and the weaknesses of the available NDT techniques. However, further research is necessary and of current interest, mainly if applied to innovative, sustainable composites recently developed. Despite proving NDT’s applicability to more sustainable bio-based composite, this study also provides crucial information on their mechanical response against loads typical in in-service conditions, like impact loads.

This present paper experimentally investigates the impact response of epoxy resin and bio-based epoxy resin composite laminates. Impact tests at increasing energies have been carried out with a drop-tower testing machine up to perforation and repeated low-energy impact tests. The residual strength and the damage tolerance of the impacted laminates have been assessed by monitoring the damage parameter Damage Index [30] and analysing the resonance frequency variation with the Impulse Excitation Technique (IET). This work shows that the resonance frequency of the damaged plates can be used to assess the damage progression of the laminates. Indeed, the Damage Index values, used in the literature to evaluate the damage progression of thick and thin composite laminates, have been correlated with the frequency values acquired with the IET technique, showing a good correlation.

## 2. Materials and Methods

### 2.1. Materials

Composite materials made of carbon fibres were fabricated using two different epoxy resins: a conventional, commercially available epoxy resin IN2 from EasyComposite Ltd. (Stoke-on-Trent, UK) [31], and a novel epoxy resin with 31% bio-content, named IB2, also from EasyComposite Ltd. (Stoke-on-Trent, UK) [32]. The bio-content in the bio-resin included glycerol derived from plants, which replaced conventional petroleum-derived propylene. The epichlorohydrin component of the bio-based epoxy resin is synthesised using renewable plant-based glycerol instead of the traditional petroleum-derived propylene. While the technical datasheet does not specify the exact plant sources for glycerol extraction, Gerber et al. [33] suggested that crop plants are the primary source. Furthermore, Miyuranga et al. [34] explained the versatility of glycerol extraction, demonstrating its derivation from various plant oils, including soybean oil. Finally, the technical information on IB2 resin [32] reported that the bio-based resin, IB2, presented a carbon footprint that was, on average, 40% less than the IN2 resin system.

Pyrofil TR30S 3k was the fabric chosen for this activity. It is produced by Mitsubishi Corporation (Tokyo, Japan). The real weight of the fibres is 210 g. The fabric has a 2 × 2 carbon twill weave. It has 3000 filaments per row (3 k). The warp density is 5.4 per centimetre. The weft density is 5.1 per centimetre. A polyester pre-coating is applied to the fabric to help the fabric handle the fibres during the fabrication.

Laminates were produced using eight layers of vacuum infusion. The resins were degassed under vacuum before infusion and cured for 24 h at room temperature. After that, IN2 resin laminates underwent a post-curing in an oven at 100 °C for 3 h. IB2 resin laminates were post-cured at 80 °C for 8 h. The curing processes were carried out as defined in the datasheet to ensure a total polymerisation of the resins. The composite plates presented a final thickness of 2.0 ± 0.1 mm with a final fibre volume fraction of 51% ± 1%. A preliminary study on the quasi-static properties of these two laminates has been carried out [12]. The summary of the mechanical properties conducted at room temperature (laminate moduli and strengths) is reported in Table 1.

### 2.2. Out-of-Plane Impact Tests

Impact tests were performed to evaluate the response of the composite laminates to out-of-plane impacts. These tests were carried out using the ASTM D5628 Standard [39]. The impact tests were performed using the FractoVIS Plus (Instron, Norwood, MA, USA) free-fall drop dart testing apparatus.

Squared specimens, 100 × 100 mm, were used for this activity. The FractoVIS uses a 76 mm ring clamping system to apply 8 bar pressure on the laminate, preventing specimen slippage during impact. The testing apparatus has an anti-rebound system to avoid multiple impacts on the specimens.

The impact energy was controlled by adjusting the velocity of the dart at the impact. The tests were carried out by keeping a constant total falling mass of 15.89 kg. The impacting dart presents a cylindrical, hemispherical shape with a diameter of 20 mm. Preliminary tests were conducted to detect the perforation threshold of the epoxy and bio-based laminate. Then, four levels were adopted to highlight a potentially different behaviour related to the adopted resin system. In particular, 15 J, 20 J and 30 J impact energies were set and verified using the velocity at impact. Further, low-energy repeated impacts, 5 J and 7 J were used to assess the energy absorption capabilities of the composite laminates made with the two resins. At least three repetitions were performed for each impact energy and material.

To measure the impact force, a piezoelectric load cell was positioned at the upper end of the dart, and load data were collected at a frequency of 1 MHz. The impact velocity was measured through a photocell.

It is worth mentioning that this apparatus also acts as the trigger for the load data acquisition process. The displacement of the dart during the impact event was calculated by double integrating the acceleration data, a(t), obtained by applying Newton’s law, as outlined in Equation (1). In the integration process, the velocity at impact was summed to the velocity obtained after the first integration to consider the velocity at the beginning of the impact event. Finally, the velocity was integrated to obtain the displacement, as clarified in the ASTM D5628 Standard [39].

(1) $$a\left(t\right)=g-\frac{F\left(t\right)}{m}$$

In Equation (1), F(t) is the time history of the force signal acquired by the load cell at the time t, m is the mass of the falling object and g is the gravitational constant. The energy E_a_ absorbed by the specimen during the impact corresponds to the area under the obtained force–displacement curve.

To monitor the progression of damage in composite laminates under repeated impacts, a Damage Index, introduced by Belingardi et al. [30], was employed in this work. This index, namely DI, is defined in Equation (2) as follows:

(2) $$DI=\frac{E_{a}}{E_{i}}\;\frac{s_{max}}{s_{i}}$$

Here, E_a_ represents the absorbed energy during the repeated impacts and E_i_ is the total impact energy (contributes to the kinetic energy computed with the velocity when the dart touches the specimens, and the potential energy related to the displacement experienced by the specimen) during the single repeated tests. Similarly, s_max_ and s_i,_ respectively, stand for the maximum displacement of the laminate during the repeated impacts and the maximum displacement in the perforation impact test. The DI ranges between 0 and 1, indicating the extent of damage sustained by the specimen, from undamaged to fully damaged. Therefore, this index serves as a tool to monitor damage propagation throughout the repeated impact test campaign. The elastic properties of the laminates were also assessed through the Impulse Excitation Technique (IET) during the repeated impact tests to compare the DI index to a physical non-destructive analysis.

### 2.3. Impulse Excitation Technique

The elastic properties of epoxy-based and bio-based resin composites were assessed using the Impulse Excitation Technique (IET) [40]. This method enables the specific characterisation of the elastic response of the impacted plates and evaluates their damage post-impact in repeated impact tests.

**Figure 1:**
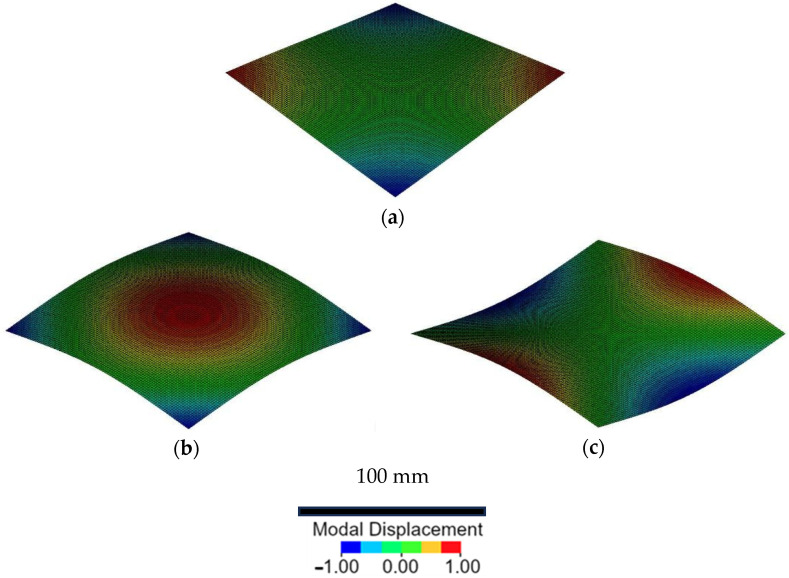
Mode shapes for the assessment of the elastic properties: (**a**) torsional mode, (**b**) O mode, (**c**) X mode.

In this study, the IET was employed to compare the material properties of the two composites. It was assumed that the elastic properties in the two principal directions were equivalent and that the plate was perfectly square with its in-plane material principal directions aligned to the plate edges. Under these assumptions, the equation $\frac{a}{b}={\left(\frac{E_{11}}{E_{22}}\right)}^{\frac{1}{4}}$ was satisfied, where a and b are the plate dimensions, and E_11_ and E_22_ represent the in-plane Young’s moduli [41]. Consequently, we decided to use the torsional, O (ring) and X modes to determine the elastic properties of composite plates. Figure 1 illustrates the considered torsional (Figure 1a), O (Figure 1b) and X modes (Figure 1c). The red colours indicate the higher displacements of these vibrational modes.

**Figure 2:**
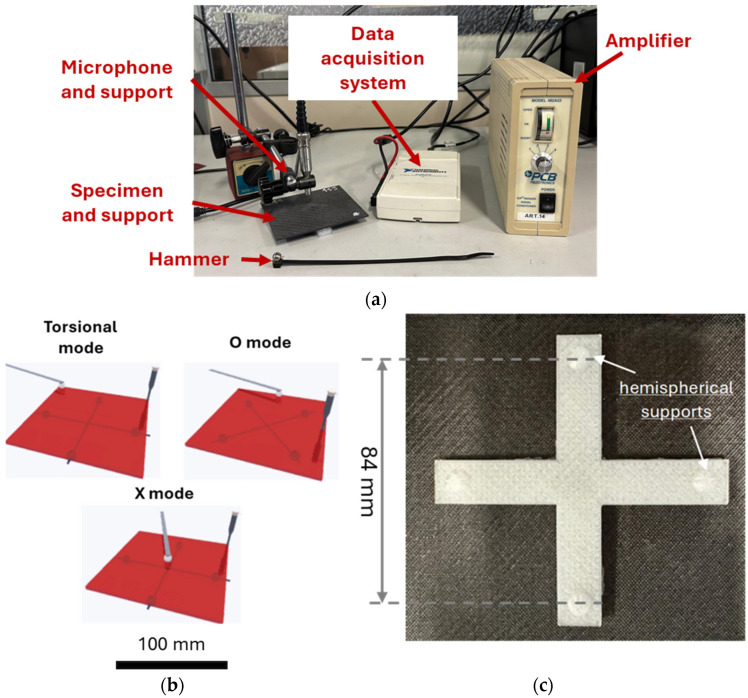
**(a**) Testing apparatus and data acquisition system, (**b**) positions of the specimens and microphones for the three investigated modes, (**c**) base of the specimens.

Figure 2 shows the IET system that has been used to assess the frequencies of undamaged and damaged plates. As illustrated in Figure 2a, the plates under investigation were supported on a base, and the microphone was opportunely positioned to detect a specific mode. The microphone signal was amplified and recorded using a data acquisition board, then analysed with Fourier Transform-based software, Buzz-O-Sonic (BuzzMac International, Portland, OR, USA) to evaluate the frequency response. The acquisition board, the NI USB-6210 system (National Instruments, Austin, TX, USA), was used for analog-to-digital conversion of the amplified signal. The position of the microphone supports and impact points where the hammer struck the plates allowed the excitation of a specific mode of vibration. In particular, the configurations for the torsional, O and X modes are shown in Figure 2b. These conditions are defined in the Software manual Buzz-O-Sonic [42]. The support is shown in Figure 2c. The hemispherical supports allowed the limit of the contact region, which should be as small as possible. The plate was moreover supported where nodal points were expected for a specific mode of vibration. In this way, the investigated mode was isolated, with a consequent limited influence of the other modes on the acquired signal. The distance between the sphere centres was 0.84 of the total length of the specimen, i.e., 84 mm.

Following the methodology proposed by McIntyre and Woodhouse [41], the in-plane shear modulus G_12_ can be calculated from the torsional mode as follows:

(3) $$G_{12}=0.822\frac{f_{T}^{2}\cdot \rho \cdot a^{4}}{h^{2}}$$

where f_T_ is the measured torsional frequency, ρ the material density, and h is the plate thickness.

The in-plane Young’s moduli, E_11_ and E_22_, and Poisson’s ratio, ν_12_, can be obtained from the O and X modes. The approximated formulas for the O and X frequencies were derived by McIntyre and Woodhouse [41] as follows:

(4) $$\begin{array}{c} f_{O}^{2}≅\frac{h^{2}}{\rho \cdot a^{4}}\left(13\cdot \sqrt{D_{11}\cdot D_{22}}+4.4\cdot D_{12}\right)\\ f_{X}^{2}≅\frac{h^{2}}{\rho \cdot a^{4}}\left(13\cdot \sqrt{D_{11}\cdot D_{22}}-4.4\cdot D_{12}\right) \end{array}$$

where:

(5) $$\begin{array}{c} D_{11}=\frac{E_{11}}{12\cdot \left(1-\nu_{12}\nu_{21}\right)}\\ D_{22}=\frac{E_{22}}{12\cdot \left(1-\nu_{12}\nu_{21}\right)}\\ D_{12}=2\cdot \nu_{12}\cdot D_{22}=2\cdot \nu_{21}\cdot D_{11} \end{array}$$

Considering that the elastic properties of the two principal directions were equal, we computed D11, D22 and D12 as follows:

(6) $$\begin{array}{c} D_{11}=D_{22}=\left(f_{0}^{2}+f_{X}^{2}\right)\frac{\rho \cdot a^{4}}{26\cdot h^{2}}\\ D_{12}=\left(f_{0}^{2}-f_{X}^{2}\right)\frac{\rho \cdot a^{4}}{8.8\cdot h^{2}} \end{array}$$

From this, Young’s moduli and Poisson’s ratios were calculated using Equation (5).

## 3. Results and Discussion

### 3.1. Perforation Tests

**Figure 3:**
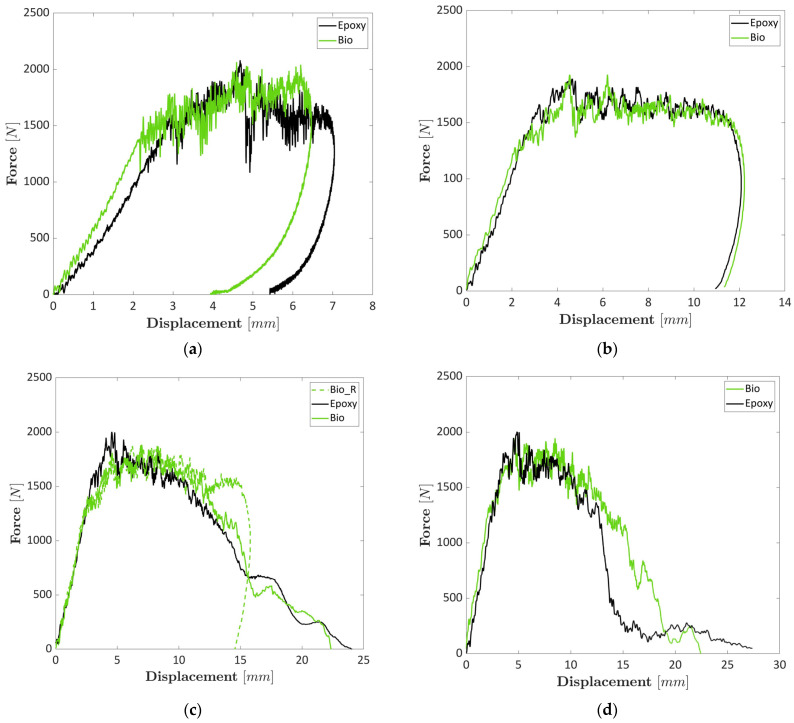
Impact tests conducted at 7 J (**a**), 15 J (**b**), 20 J (**c**) and 30 J (**d**).

**Figure 4:**
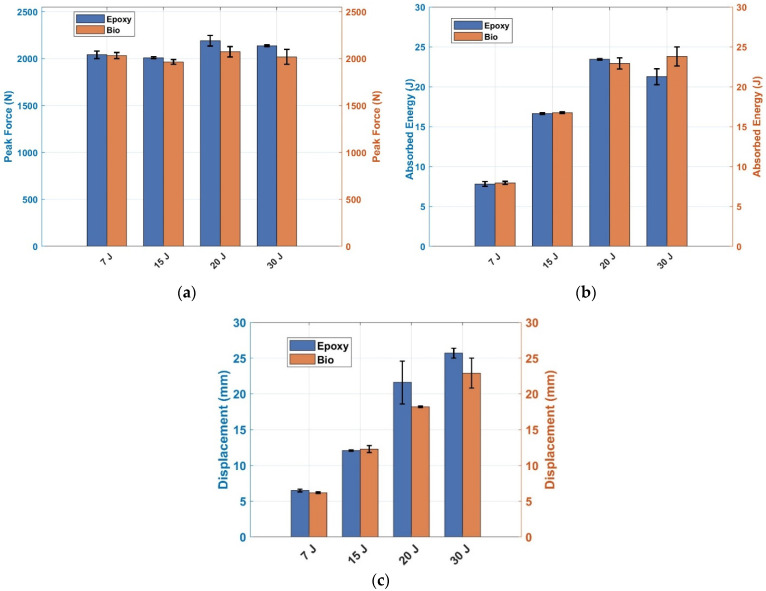
(**a**) Peak forces of bio and epoxy composites; (**b**) absorbed energy of bio and epoxy composites; (**c**) maximum displacements of the composite laminates prepared with bio and epoxy resins.

Figure 3 shows representative force–displacement curves at different increasing impact energies of 8.6 J, 16.5 J, 23.5 J and 33.5 J. Representative curves are shown due to the limited scatter between the curves of the same set of samples as it is indicated by the error bars of the charts in Figure 4. The impact energy took into account the energy computed with the velocity when the impact started (namely 7, 15, 20 and 30 J) plus the energy added due to the displacement of the dart within the specimen during the tests. The force–displacement curves of laminates prepared with both epoxy and bio-epoxy resins presented a very similar behaviour in all four analysed cases with a slight difference at higher impact energy, 20 and 30 J, when the perforation of the composite laminates and the falling dart experienced a rebound due to the elastic energy of the specimen. The rebound was visible by analysing the force–displacement curves, which ended with a negative displacement. Impacts carried out at 7 and 15 J, Figure 3a and Figure 3b, respectively, could not perforate the specimens. However, at 15 J, the displacement was almost doubled compared to the impact at 7 J, while the maximum forces remained practically constant. At 20 J, Figure 3c, two curves over three are shown for the specimens prepared with the bio-based epoxy resin because two different behaviours were obtained. For this reason, a bio curve representative of the specimens that presented the rebound is also reported in Figure 3c. Indeed, the composite laminates prepared with bio-resin experienced a rebound in two out of three cases. This behaviour indicated that these specimens still present a residual elastic energy compared to the laminates prepared with the conventional epoxy resin. An explanation of this property can be related to the higher displacement and deformation that the bio-resin exhibits over the conventional resin, as shown in a preliminary work of the same authors [12], especially in flexural tests. The higher ductile behaviour of the resin explains why the composite prepared with bio-resin can bear a higher load before perforation. Figure 3d illustrates that 30 J was enough to perforate both types of laminates. However, the specimens prepared with bio-resin presented a final drop of the load at 20 mm, while the epoxy-based laminates presented a drop close to a 15 mm load drop. These different drops led to a higher value of the absorbed energy for specimens prepared with bio-resin.

Figure 4a–c summarise the experimental activity shown in Figure 3. Figure 4a and Figure 4b show the values of the forces and the absorbed energy, respectively, for composite laminates prepared with bio-based resin and epoxy resin, respectively. The maximum force, around 2000 N, did not change significantly for any of the two types of specimens and the four analysed impact energies. This trend can be explained by considering that the fibre contents were similar. As shown in Figure 3, the damage was also triggered in laminates impacted at 7 J, as shown in the load–displacement curves. Indeed, load–displacement curves in Figure 3a do not show a perfectly elastic rebound since the displacement did not reach 0 mm after the impact. Thus, the values were similar, having the same number of layers for both conventional epoxy resin and bio-resin. Contrarily, comparing absorbed energies at different impact energy levels presented increasing values. Similar values at 7 and 15 J, where both specimens experienced a rebound, were obtained. Comparable values were also obtained at 20 J. However, the comparison is unfair in this case since two specimens out of 3 for the laminate prepared with bio-resin experienced a rebound.

In contrast, complete perforation was obtained in all cases for the specimen prepared with the conventional epoxy resin. Finally, the values of the absorbed energy at 30 J showed that the specimens prepared with bio-resin absorbed higher energy. Bio-based specimens bore a higher load before fracture at around 15 mm, while epoxy-based laminates exhibited a clear drop before failure, as shown in Figure 3d. The higher bearing capacity before failure can be related to the different matrices that have been used and the higher elongation that the bio-polymer displays. Boursier Niutta et al. [12] showed a similar behaviour of the same laminates for quasi-static tests. In particular, the bio-based laminates exhibited higher flexural strain than the epoxy-based ones.

Ultimate displacements are shown in Figure 4c. At 7 and 15 J, the ultimate displacements of both specimens prepared with the two resins were the same due to the rebound. At 20 J, the bio-based laminate presented lower displacement values due to the rebound of the specimens prepared with bio-resins. At 30 J, the displacement values of the specimens prepared with epoxy resin looked larger. However, the main significant drop occurred before 15 mm, and then the load remained constant between 100 and 200 N before going to 0 N. The specimens prepared with bio-resin presented a significant drop in the load at around 20 mm, which allowed them to absorb more energy during failure.

### 3.2. Repeated Impact Tests

**Figure 5:**
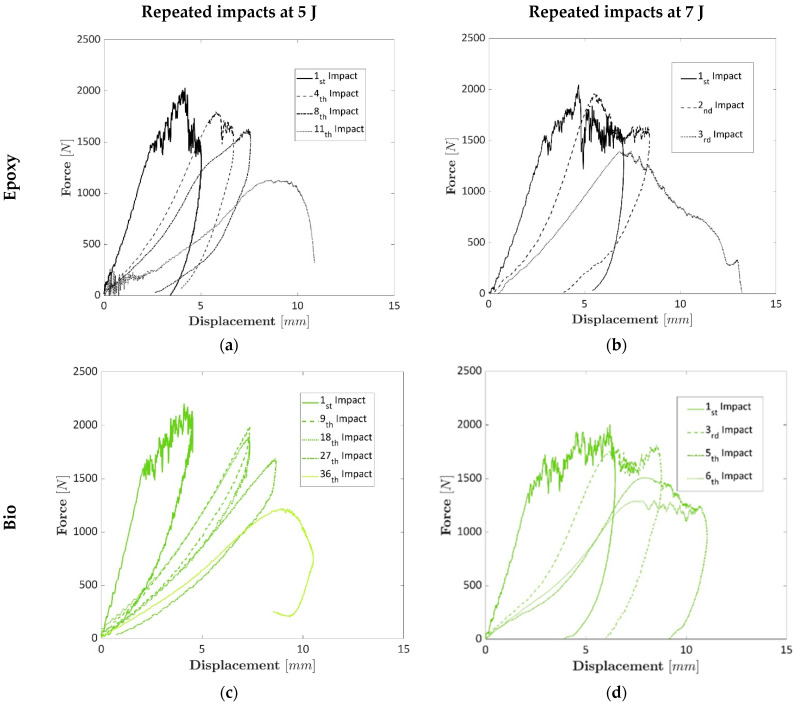
(**a**) Representative load–displacement curves for the laminates prepared with the epoxy resin at 5 J; (**b**) Representative load–displacement curves for the laminates prepared with the epoxy resin at 7 J; (**c**) Representative load–displacement curves for the laminates prepared with the bio-resin at 5 J; (**d**) Representative load–displacement curves for the laminates prepared with the bio-resin at 7 J.

Repeated tests were carried out to assess whether the specimens prepared with bio-resin presented higher resistance at repeated impacts due to the larger displacements they presented in the perforation tests. Figure 5a and Figure 5b show the repeated impact tests for the epoxy-based laminates at 5 J and 7 J, respectively. Figure 5c and Figure 5d display the repeated impact tests for the epoxy-based laminates at 5 J and 7 J, respectively. Different representative curves were chosen for each laminate due to the different numbers of obtained repetitions to highlight the damage progression among the different impacts. The damage progression can be shown by the decreased stiffness value (initial trend of the curves) and the increased displacement during repeated tests. The repeated impacts showed that at 5 J, the epoxy-based laminates bore 11 impacts, while the bio-based laminates reached 36 impacts. At 7 J, the conventional epoxy-based laminates bore three impacts, while the composite bio-based resin failed after six repeated impacts. The most significant differences between laminates prepared with epoxy and bio-resins can be found in the first drop of mechanical properties when the first peak was reached at both 5 and 7 J and the rebound of the dart that was identified with the values of the negative displacements in the load–displacement curves. The first curve of the epoxy laminates impacted at 5 J and 7 J showed a slight drop before 5 mm, Figure 5a,b, that was not present in the specimens prepared with bio-resin, Figure 5c,d. During the damage progression, the laminates prepared with bio-based resin exhibited higher force values sustained over more significant displacements, indicating superior residual elastic properties and lower damage levels than those made with epoxy resin under the same number of impacts. This highlights the ability of bio-based resins to maintain a more consistent and elevated load capacity over repeated impacts. This trend is visible when comparing the first and the last curves of epoxy and bio-based epoxy at both 5 J and 7 J. Contrarily, the curves of the epoxy-based laminates present lower values of the forces after the maximum peak, which leads to a lower absorbed energy. Finally, the bio-based composite plates presented a higher rebound up to the failure, which means a higher residual energy. The meaning of this behaviour is attributed to the higher deformation that the epoxy bio-based resin can offer before failure, with a consequent higher number of impacts for the resin prepared with the bio-based resin.

### 3.3. Damage Index

**Figure 6:**
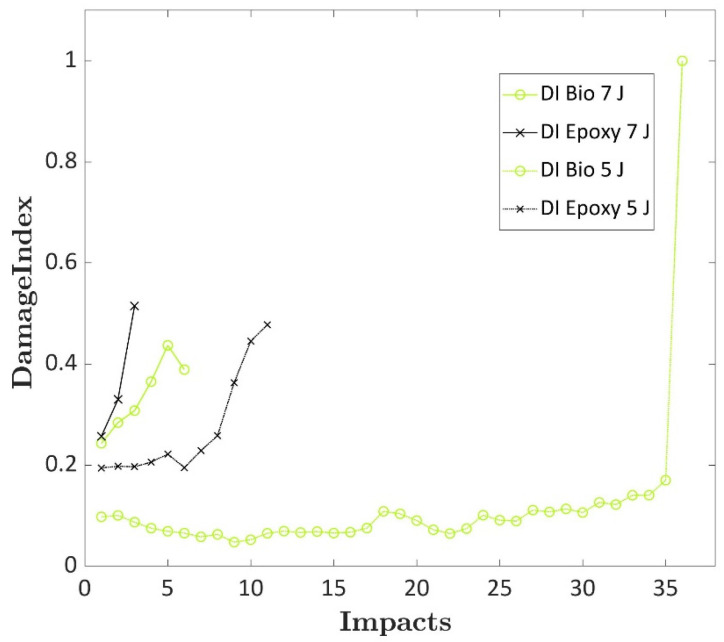
DI computed for the epoxy and bio laminates at impact energies of 5 J and 7 J.

The DI was computed to analyse the damage progression of the epoxy and bio-based laminates to repeated impacts, and the curves are shown in Figure 6. Usually, the last impact that is carried out on the laminates presents low absorbed energy due to the limited residual strength or a very high energy based on the extensive displacement that the specimens can reach after failure with a force increase due to the friction of the dart and the fractured specimens. A DI of 1 was assigned to the last impact for this latter case. The DI computed for the laminates impacted at 5 J showed that both laminates presented very different DI values due to the different damage progression depicted for the two types of specimens. In particular, the DI of the biospecimen impacted at 5 J presented an initial value that was half the epoxy specimen value when impacted at the same energy. Then, when damages started to increase, the DI values significantly increased for both laminates before failure. This increase was more evident for the epoxy specimen, where the DI value rose from an average value of 0.2 to a maximum value of 0.5.

On the other hand, the biospecimens increased from an average value of 0.1 to 0.17 before the complete failure. A different behaviour was observed for specimens impacted at 7 J. The DI of bio and epoxy specimens impacted at 7 J showed larger values than those impacted at 5 J. The DI presented a value close to 0.25, and the biospecimens, which failed after six repeated impacts, presented slightly lower values. Moreover, the damage quickly progressed at this energy level, and the DI did not show a plateau visible for the specimens impacted at 5 J. The trend of the epoxy specimen was sharper compared to the bio-based specimen that failed after the double numbers of impact. Thus, the DI can detect the higher damages caused by higher energy levels and the different damage progressions in the composite that present different matrices due to their elastic properties. In the following section, the same analysis was carried out using the IET technique. The DI trend can be considered as a reference since the DI can effectively detect the damage progression of composite laminates, as reported in the literature [2].

### 3.4. IET

**Figure 7:**
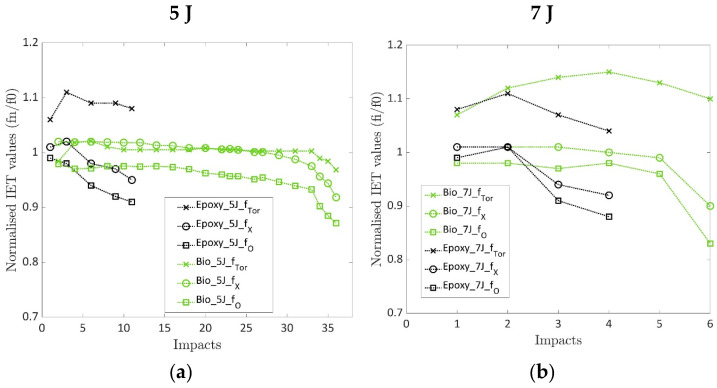
(**a**) Normalised IET computed for the epoxy and bio laminates at an impact energy of 5 J; (**b**) Normalised IET computed for the epoxy and bio laminates at an impact energy of 7 J.

Figure 7a,b show the normalised values of the acquired resonance frequencies for the repeated impacts. The normalised values were computed using the ratio $\frac{f_{n}}{f_{0}}$, where f_n_ is the frequency of the repeated impact and f_0_ is the initial frequency of the undamaged specimen, to show whether the frequency decreased or increased while damage progressed. As reported in Section 2.3, the torsional mode, the O mode and the ring modes, namely $f_{tor},$ $f_{O}$ and $f_{X},$ were computed and were reported for both the laminates under repeated impact at 5 and 7 J. The green curves represent the bio-based laminates in Figure 7a,b, while the black curves represent epoxy. The torsional mode frequency for both laminates prepared with conventional and bio-based resins presented an increasing value or values after the first impact that then decreased before the failure. The increase in the frequency of the $f_{tor}$ mode was expected. As shown in Figure 1, this modal shape has nodal zones where the damage occurred. The presence of damage in areas that did not participate in the vibration was not detectable. Moreover, the presence of damage prevented the vibrations, thus extending the nodal zone and causing an increase in resonant frequency $f_{tor}$. The same phenomenon happened for $f_{X}$ in the case of impacts at 5 J, and it could be due to the same reason. On the contrary, the damage induced by the 7 J impacts extended into the non-nodal region, thus participating in the mechanical vibration and causing a decrease in the resonant frequency. On the other hand, the $f_{O}$ Mode showed a decreasing trend for both laminates impacted at 5 J and 7 J. This decrement was expected since the maximum modal displacement occurred at the location of the damage, as shown in Figure 1, and no nodal points were present in the damaged zone. The values of the normalised frequencies at first for the impacts carried out at 5 and 7 J did not show significant differences. This can be attributed to the limited size of the damage that does not influence the vibration of the specimens. Contrarily, the final values of the normalised frequency for epoxy and bio-based epoxy laminates were the same at the last impact. This result is very positive since the specimens reported similar failure surfaces, as noted in Section 3.6, and the final value could be used to assess the damage level of composite laminates if a complete characterisation of the materials is available. By considering the final values at rupture, the bio-resin specimens presented normalised frequency values higher than the ones prepared with the conventional epoxy resin. This behaviour depends on the larger damaged area these specimens undergo, as explained in Section 3.6. Further, the normalised IET values presented a plateau area and a decreasing area during the steady damage progression and before failure, similar to the IET index.

### 3.5. Comparisons Between IET and DI Index

**Figure 8:**
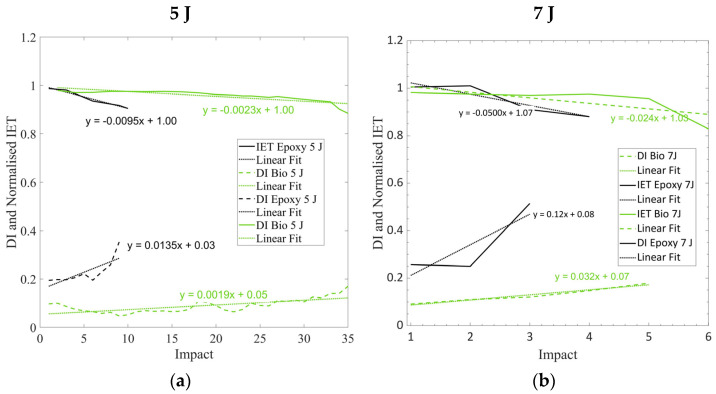
(**a**) Comparison between normalised IET and DI computed for the epoxy and bio laminates at an impact energy of 5 J; (**b**) Comparison between normalised IET and DI computed for the epoxy and bio laminates at an impact energy of 5 J.

The comparison between the DI values and normalised frequency (f_O_) is reported in Figure 8a and Figure 8b for 5 J and 7 J impacts, respectively. The dashed lines report the DI values, while the solid lines report the normalised IET values. A linear regression of both the DI and normalised IET, with respect to the number of impacts, was carried out and is reported with dot lines. Only DI values up to 0.4 were chosen to consider the linear regression similar to what was undertaken by [30]. As shown in Section 3.3 and Section 3.4, the normalised IET showed a decreasing trend, while the DI showed an increasing trend as the damage progressed. The interpolating equations are also reported in Figure 8a,b. The DI and Normalised IET presented very similar slopes, especially for the specimens impacted at 5 J, which presented a steady damage progression before failure.

### 3.6. Fracture Surfaces

**Figure 9:**
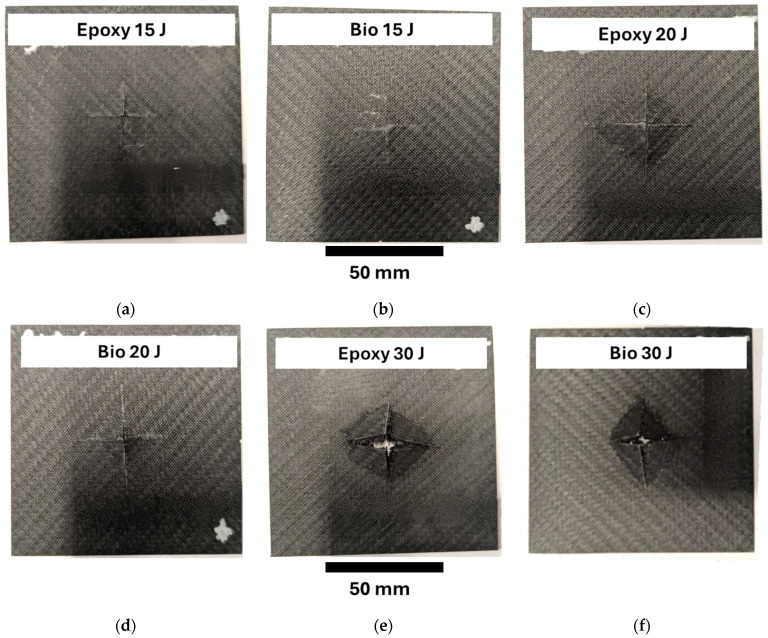
Fracture surfaces for the specimens impacted at 15 J, 20 J and 30 J: (**a**) Bio 15 J, (**b**) Epoxy 15 J, (**c**) Bio 20 J, (**d**) Epoxy 20 J, (**e**) Bio 30 J, (**f**) Epoxy 30 J.

Figure 9 displays the representative fracture surfaces of the perforation tests at 15 J, 20 J and 30 J for the plates prepared with epoxy and bio-resins. The fracture surface at 7.5 J has not been reported since tiny indentations were detected. The surfaces of the specimens impacted at 15 J, Figure 9a,b, showed that the plate prepared with epoxy resin presented split cracks in two directions, while the specimens prepared with bio-based resin presented closed superficial cracks. The epoxy and bio-based impacted specimens at 20 J, Figure 9c,d, showed that the damaged area presented similar sizes. However, the specimens prepared with epoxy resin presented an evident open indentation related to the failure of the laminates. On the other hand, the biospecimens experienced a rebound at 20 J, as reported in Section 3.1. Finally, the specimens impacted at 30 J showed that both specimens prepared with epoxy and bio-based resins, Figure 9e,f, presented similar failure surfaces, sizes, and opened cracks. However, the open patches were smaller and less open for the specimens prepared for the bio-based resin.

**Figure 10:**
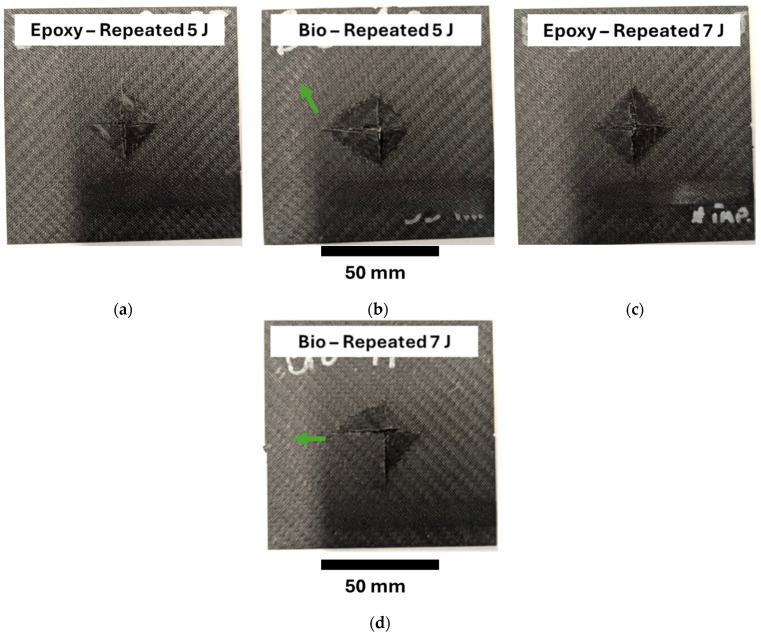
Fracture surfaces for the specimens subjected to repeated impact tests at 5 J and 7 J: (**a**) Epoxy 5 J, (**b**) Bio 5 J, (**c**) Epoxy 7 J, (**d**) Bio 7 J.

Figure 10 reports the specimens subjected to repeated impact tests at 5 J, Figure 10a and Figure 10b, and at 7 J, Figure 10c and Figure 10d, respectively, for plates prepared with epoxy and bio-resins. Figure 10 shows that the plates prepared with the epoxy resin and impacted at 5 J and 7 J presented similar fracture surfaces. The size of the damaged area and the shape were symmetric and quite close to the specimens impacted at 30 J, where complete perforation occurred. On the other hand, the plates prepared with the bio-based resin and impacted at 5 J and 7 J presented similar damage areas that were slightly longer on the left side, Figure 10b,c. This indentation was the result of the higher number of impacts that these specimens bore. The damaged area was slightly higher for bio-based due to the higher displacement that these specimens bore. Likely, the ductile behaviour of the plates prepared with bio-based resin triggers a different damage mechanism, and the interlaminar crack propagates before the final fragile failure. This behaviour involved a wider part of the plates in the damage progression. Indeed, the plates presented a tiny indentation close to the circular support, as shown by the green arrow. The frequencies computed throughout the IET also showed that this technique could detect greater damage. Indeed, the specimens impacted at 5 J presented a final value of 0.86 and 0.90 for the specimens prepared with bio and epoxy resins, respectively. The final values for specimens impacted at 7 J were 0.82 and 0.88, respectively, for the bio and epoxy resins. Thus, the plates with greater damage, the biospecimens, presented a higher drop in frequency.

**Figure 11:**
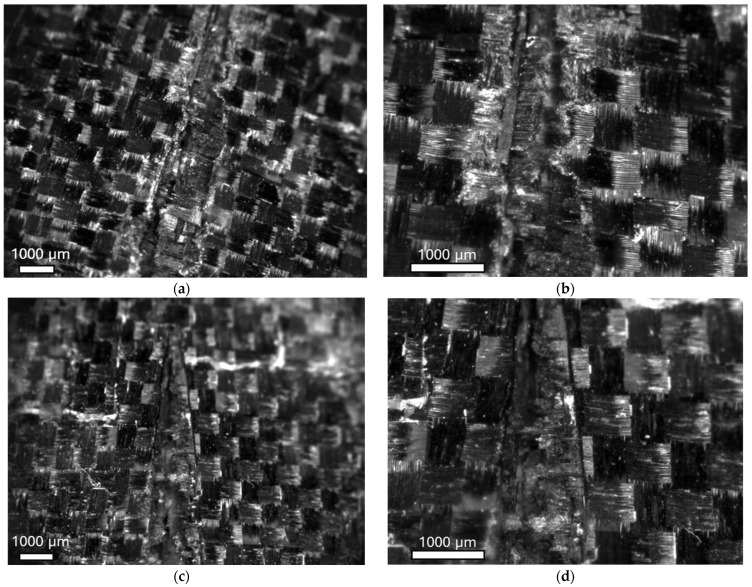
Micrography of the epoxy specimens at two different magnifications, 1× (**a**) and 200× (**b**); Micrography of the epoxy specimens at two different magnifications, 1× (**c**) and 200× (**d**).

Figure 11a,b report a micrograph of the fracture surfaces of the epoxy laminate at a point where the crack of the laminate failed at two different magnifications (1× and 200×). Thus, an evident crack was visible in the centre of the specimen. Figure 11c,d present micrographs of the fracture surfaces of the bio laminate at the same location at two different magnifications (1× and 200×). This analysis was carried out on specimens impacted at 30 J and are reported as representative surfaces since all the tests led to perforation and presented similar failure surfaces. For both specimens, good adhesion between the matrix and fibres was observed since no evident areas were pulled out from the matrix observed.

## 4. Conclusions

The impact behaviour of composite laminates prepared with a conventional and a sustainable epoxy resin was investigated. Furthermore, the use of the Impulse Excitation Technique (IET) to assess the damage progression was thoroughly verified.

The experimental activity showed that composite plates prepared with the bio epoxy resin absorbed higher energy at perforation due to the higher ductility of the resin. The composite plates prepared with bio-resin bore the maximum force for a more extended displacement compared with the plates prepared with the conventional epoxy resin.

Repeated impact tests showed that the composite plates prepared with bio-resin presented a lower damage progression related to the higher ductility of the bio-based resin.

The Damage Index, widely adopted in the literature for assessing the damage progression of thin and thick composite materials, was used to verify the effectiveness of the IET technique in physically quantifying the damage progression. The experimental results showed that the frequencies measured with IET detected the damage progression of composite plates and composites with a wider damaged area present and a higher drop in the frequency. The results obtained by IET techniques were in good agreement with the values of the DI, thus confirming the effectiveness of this technique as a damage indicator. The IET effectively measures global elastic properties but presents limitations in assessing composite laminate damage if the adopted vibrational modes are not in the area of higher nodal displacement. Furthermore, it shows low sensitivity to minor defects and a lack of differentiation between damage types.

## Figures and Tables

**Table 1:** Mechanical properties of the investigated composites [12].

	IN2 Carbon Composite	IB2 Carbon Composite	Standard (Velocity of the Test)
Tensile Modulus [GPa]	57.7 (±0.7)	54.8 (±0.1)	ASTM D3039 [35] (2 mm/min)
Tensile Strength [MPa]	715.5 (±4.5)	650 (±5.0)	
Flexural Modulus [GPa]	41.7 (±4)	39.4 (±0.1)	ASTM D790 [36] (1 mm/min)
Flexural Strength [MPa]	658 (±1.0)	627 (±16.5)	
Compression Modulus [GPa]	42.6 (±1.4)	45.1 (±1.5)	ASTM D3410 [37] (1.3 mm/min)
Compression strength [MPa]	448.5 (±7.5)	462 (±11.0)	
Shear Modulus [GPa]	4.1 (±0.1)	3.8 (±0.1)	ASTM D5379 [38] (2 mm/min)
Shear Strength [MPa]	58.1 (±0.5)	60.4 (±1.4)	

## Data Availability

The original contributions presented in the study are included in the article, further inquiries can be directed to the corresponding authors.
